# Supplementary material for: Patterns of Intron Gain and Loss in Fungi
Source: PLoS Biol. 2004 Nov 30;2(12):e422. doi: 10.1371/journal.pbio.0020422 (PMC532390; doi:10.1371/journal.pbio.0020422)
Supplement: Table S1 — Also available at http://genes.mit.edu/NielsenEtAl/. (4.3 MB ZIP). [file pbio.0020422.st001.zip › NielsenEtAl/html/858.html]

AN1358.1.NCU04600.1.MG01351.1.FG10239.1


```
 CLUSTAL W (1.82) Multiple Sequence Alignments - Introns Inserted


Sequence 1: NCU04600.1	439 aa
Sequence 2: FG10239.1	430 aa
Sequence 3: AN1358.1	305 aa
Sequence 4: MG01351.1	451 aa
Alignment Length: 456 aa
Number Identitical Residues: 215 aa
Alignment Score (without introns) 10819


MG01351.1 	MGQTLSEPVVEK0ASARGEDDRFLYGVSAMQGWRISMEDAHTTVLDLLANDAEAAKQHKG
NCU04600.1	MGQTLSEPVVEK0ASATGGDERLIYGVSAMQGWRISMEDAHTTVLDLLANNPKEAKDHSQ
FG10239.1 	MGQTLSEPVVEK0TSEKGEDERLIYGVSAMQGWRISMEDAHTTVLDLDT-----AKTHDS
AN1358.1  	------------~-----------------------MEDAHAAVLDLQAKQSGSNDQPTD
          	                                    *****::**** :...   .    

MG01351.1 	---KLAFFGVFDGHGGDKVALFSGDNIHKIVQNQDTFKSGNYEQALKDGFLATDRAILND
NCU04600.1	---KLSFFGVFDGHGGDKVALFAGANIHDIIAKQDTFKTGNYEQALKDGFLATDRAILND
FG10239.1 	---KLSFFGVFDGHGGDKVALFTGQNIHNIIFKQDTFKSGDYAQGLKDGFLATDRAILND
AN1358.1  	PDRRLAFFGVYDGHGGDKVALFAGENVHKIVAKQETFLKGDIEQALKDGFLATDRAILED
          	.. :*:****:***********:* *:*.*: :*:** .*:  *.*************:*

MG01351.1 	1PKYEDEVSGCTACVGLLTDDKIYI0ANAGDSRSVLGVKGRAKPLSFDHKPQNEG1EKAR
NCU04600.1	1PKYEEEVSGCTACVGLITDEKIFV0ANAGDSRSVLGVKGRAKPLSFDHKPQNEG1EKAR
FG10239.1 	1PKYEEEVSGCTACVSLIAGNKLYV0ANAGDSRGVLGIKGRAKPLSQDHKPQLEN1EKNR
AN1358.1  	1PKYEEEVSGCTAAVSIISKKKIWV0ANAGDSRSVLGVKGRAKPLSFDHKPQNEG1EKAR
          	 ****:*******.*.::: .*::: *******.***:******** ***** *. ** *

MG01351.1 	ITAAGGFVDFGRVNGNLALSRAIGDFEFKKSAELAPEQQIVTAYPDVVVHDMGDDDEFLV
NCU04600.1	ITAAGGFVDFGRVNGNLALSRAIGDFEFKKSAELAPEQQIVTAYPDVMVHDLADDDEFLV
FG10239.1 	ITAAGGFVDFGRVNGNLALSRAIGDFEFKKSAELSPENQIVTAYPDVEQHDLTDEDEFLV
AN1358.1  	ISAAGGFVDFGRVNGNLALSRAIGDFEFKKSPELSPEQQIVTAYPDVTVHELTEDDEFLV
          	*:*****************************.**:**:*********  *:: ::*****

MG01351.1 	IACDG1IWDCQSSQAVVEFVRRGIAAKQALEKICENMMDNCLASNSETGGVGCDNMTMII
NCU04600.1	LACDG1IWDCQSSQAVVEFVRRGIAAKQDLDKICENMMDNCLASNSETGGVGCDNMTMII
FG10239.1 	LACDG1IWDCQSSQAVVEFVRRGIAAKQELEKICENMMDNCLASNSETGGVGCDNMTMCI
AN1358.1  	IACDG1IWDCQSSQAVVEFVRRGIAAKQDLYRICENMMDNCLASNSETGGVGCDNMTMVI
          	:**** ********************** * :************************** *

MG01351.1 	IGFLRGRTKEEWYEEIARRVANGDGPCAPPEYA1EFRGPGVHHNFDDSDSGYDVDMDQSK
NCU04600.1	VGFLRGRTKEEWYEEIAKRVANGDGPCAPPEYA1EFRGPGVHHNFDDSDSGYDLEDNGNK
FG10239.1 	IGFLNGKSKDEWYEEIARRVANGDGPCAPPEYA1EFRGPGVHHNFEDSDSGYEMDPE-NK
AN1358.1  	IGLLNGKTKEEWYNQIAERVANGDGPCAPPEY-~--------------------------
          	:*:*.*::*:***::**.**************                            

MG01351.1 	GKTFGIGGYKGRVIFLGDGTEVLTDADDNAMFE--SEKQDLDSQVAKTGSSEDDGTTEDQ
NCU04600.1	GKPFGMGGYKGRIIFLGDGTEVLTDADDTEMFDNVDEDKDLASQVSKSPSSITTNDQDQK
FG10239.1 	GRSFGVGGYRGRIIFLGDGTEVLTDSDDTEMFDNADEDKDLASQVSKNPTSAEKDTAPVA
AN1358.1  	------------------------------------------------GKSLEEPTASNP
          	                                                 .*         

MG01351.1 	TNKKEGTTDGKADKGTKDASEEAEAKAENIDAAVKKDEDIKV
NCU04600.1	E--QAAVAAAAADNSTQNAKKEETSSPAKA------------
FG10239.1 	A--PGAAENASADTKPADSAKTEVKKE---------------
AN1358.1  	Y-----------------------------------------
          	
```
